# Socioeconomic and Other Social Stressors and Biomarkers of Cardiometabolic Risk in Youth: A Systematic Review of Less Studied Risk Factors

**DOI:** 10.1371/journal.pone.0064418

**Published:** 2013-05-17

**Authors:** Natalie Slopen, Elizabeth Goodman, Karestan C. Koenen, Laura D. Kubzansky

**Affiliations:** 1 Center on the Developing Child, Harvard University, Cambridge, Massachusetts, United States of America; 2 Department of Social and Behavioral Sciences, Harvard School of Public Health, Boston, Massachusetts, United States of America; 3 Massachusetts General Hospital, Center for Child and Adolescent Health Policy, Boston, Massachusetts, United States of America; 4 Department of Pediatrics, Harvard Medical School, Boston, Massachusetts, United States of America; 5 Department of Epidemiology, Columbia University Mailman School of Public Health, New York, New York, United States of America; Queensland University of Technology, Australia

## Abstract

**Background:**

Socioeconomic disadvantage and other social stressors in childhood have been linked with cardiometabolic diseases in adulthood; however the mechanisms underlying these observed associations and the timing of their emergence are unclear. The aim of this review was to evaluate research that examined relationships between socioeconomic disadvantage and other social stressors in relation to less-studied cardiometabolic risk factors among youth, including carbohydrate metabolism-related factors, lipids, and central adiposity.

**Methods:**

We searched PubMed and ISI Web of Science to identify relevant publications between 2001 and 2013.Studies were selected based on 4 criteria: (1) the study examined an association between at least one social or economic stressor and one relevant outcome prior to age 21; (2) the sample originated from a high-income country; (3) the sample was not selected based on a health condition; and (4) a central aim was to evaluate the effect of the social or economic stressor on at least one relevant outcome. Abstracts were screened and relevant publications were obtained and evaluated for inclusion criteria. We abstracted data from selected articles, summarized them by exposures and outcomes, and assigned an evidence grade.

**Results:**

Our search identified 37 publications from 31 studies. Socioeconomic disadvantage was consistently associated with greater central adiposity. Research to date does not provide clear evidence of an association between childhood stressors and lipids or carbohydrate metabolism-related factors.

**Conclusions:**

This review demonstrates a paucity of research on the relationship of socioeconomic disadvantage and other social stressors to lipid and carbohydrate metabolism-related factors in youth. Accordingly, it is not possible to form strong conclusions, particularly with regard to stressors other than socioeconomic disadvantage. Findings are used to inform priorities for future research. An improved understanding of these pathways is critical for identifying novel prevention targets and intervention opportunities to protect the long-term health of children and adolescents.

## Introduction

Cardiometabolic diseases are a leading cause of morbidity and mortality in the United States and the prevention of future cardiometabolic diseases is among the most significant public health challenges faced by contemporary society [1]. Several recent national policy statements point to childhood as a critical period for preventing cardiometabolic risk over the life course [2], . For example, a statement from the American Heart Association highlights emerging evidence that cardiometabolic physiological dysregulation begins in childhood [4], [5] and that risk factor control in children is crucial [2]. A recent statement by the American Academy of Pediatrics (AAP) further identified the importance of the early social environment in setting up risk (or resilience) trajectories, and encouraged pediatric providers to assess family or community-level risk factors that may put children at risk for experiencing toxic social stress [3]. While there is increasing urgency to identify and address determinants of early biological risk factors for adult chronic diseases, our understanding of whether and how social adversity influences cardiometabolic risk factors that emerge early in life remains somewhat limited.

Research in developmental biology has made a compelling case that early exposure to social disadvantage and toxic stress has lifelong consequences for health by virtue of biologically embedding (i.e., altered biological functioning as a result of exposure) [6], [7]. Potentially toxic social stress in childhood may arise from a range of sources such as exposure to violence at home and in the neighborhood, dysfunctional schools, personal maltreatment, household chaos, or absent or punitive parents, among others. And, children who are socioeconomically disadvantaged may be especially vulnerable to biological embedding of disease by virtue of disproportionate exposure to a multitude of stressful influences [6]. Research suggests that such exposures may play a role in early risk of chronic disease later in life, including Type 2 diabetes and cardiovascular disease (CVD) [8]–[11].

The recent AAP policy statement also highlighted the importance of identifying early physiological mechanisms through which early psychologically and physically stressful experiences, such as poverty or maltreatment, increase later risk for disease [3]. Such work is needed to inform development and assessment of interventions that can promote healthy trajectories and disrupt health-damaging trajectories before disease processes are initiated [12]. While numerous relevant studies have been conducted, it is as yet unclear as to whether consistent findings are emerging and how this work may best inform intervention. Moreover, there has been substantial research on socioeconomic disadvantage and other social stressors in relation to overweight and obesity among youth [13]–[15]; however there are other relevant cardiometabolic factors to consider. As a crucial initial step toward translating advances in developmental science into more effective interventions for reducing risk of cardiometabolic disease in adulthood [3], the aim of this review is to assess what is known about the relationship between childhood stressors and important but less-studied cardiometabolic risk factors among youth: carbohydrate metabolism-related factors, lipids, and central adiposity.

Socioeconomic disadvantage and other social stressors, defined as external conditions or events that threaten a child's wellbeing, may occur at the individual, household, or community-level. Stressful experiences may affect cardiometabolic risk through behavioral factors (e.g., unhealthy diet or inactivity), or direct physiological changes resulting from disruption of regulatory pathways. Research shows that adverse experiences are associated with of a variety of physiological changes in children [16], including increased activation of neurobiologic systems responsive to stress, such as the hypothalamic pituitary adrenal (HPA) axis, the sympathetic nervous system, and others [7], [16]–[18]. Increased activation of these systems leads to a cascade of physiological processes [7], [16] which in adults, has been linked with the development of central fat, dysregulated carbohydrate metabolism, and accumulation of blood lipids in the arterial lining, all of which accelerate chronic disease development [19]. However, to date there has been limited examination of whether childhood adversity leads to visible early dysregulation in cardiometabolic processes in youth, beyond a substantial focus on obesity. Consequently, we have a limited understanding of whether increased activation of stress-responsive systems does in fact lead to dysregulated carbohydrate metabolism, accumulation of blood lipids, or central adiposity among children.

Recent review articles report consistent evidence that early socioeconomic disadvantage and other social stressors are associated with childhood overweight and obesity, with reviews focused on family socioeconomic status (SES) [13], neighborhood characteristics [15], and psychosocial stressors [14] as exposures. This research on obesity provides a starting point for understanding the relationship between early experiences and adult chronic disease risk. However, there are other measurable aspects of cardiometabolic regulation (such as glucose metabolism, lipid profiles, or the distribution of fat) in childhood which have been shown to be strong predictors of cardiometabolic parameters into adulthood [20]–[24]. While these processes are highly inter-related (e.g., obesity is associated with elevated levels of glucose, unhealthy lipids, and central adiposity [25], [26]) they also reflect distinct cardiometabolic processes with multifactorial influences and therefore warrant separate investigation. Research on the association between socioeconomic disadvantage and other social stressors and carbohydrate metabolism-related markers, lipids, and central adiposity has not been evaluated systematically; therefore, it is yet unknown if social adversity has a detectable influence on these parameters in childhood or adolescence

To respond to recent interest and calls for greater focus on childhood origins of cardiometabolic diseases [27], we evaluated research that examines socioeconomic disadvantage and other social stressors in relation carbohydrate metabolism-related factors, lipids, and central adiposity in children or adolescents in a systematic literature review. Because of the tight clustering of the carbohydrate metabolism-related biomarkers, we expected a consistent pattern of associations of SES and other social stressors with these outcomes. In contrast, we did not expect consistent associations across lipid biomarkers since they reflect a more diverse set of physiological processes, albeit all related to CVD risk. Systematic reviews are invaluable to researchers, health care providers, and policy makers because they provide an integrated unbiased summary of existing information, and establish whether findings are consistent and can generalize across populations, settings, and differences in study design [28]. A systematic evaluation of the current findings and the quality of existing studies will aid in identifying the most fruitful directions for future research and making informed recommendations. We further consider our findings in relation to reviews of social adversity and other more studied cardiometabolic risk factors in youth. A meta-analysis of the relevant literature was not possible at this time due to the heterogeneity of studies with regard to exposure and outcome measures, study design, covariates, and samples.

## Methods

### Inclusion Criteria

We applied four inclusion criteria, informed by previous systematic reviews. First, we required that the sample originated from a high-income country, according to the World Bank (Gross National Income per capita>US$12,480 in 2011); this criteria was established because the relationship between stressors and cardiometabolic outcomes may differ in poorer countries [15]. Second, we required that the sample was not selected based on a health condition, because our primary interest was to examine these associations in healthy children and adolescents. Third, studies were required to examine an association between at least one social or economic stressor and one relevant outcome (described below) prior to age 21. We defined childhood broadly in order to include as many studies as possible. Finally, we required that evaluating the effect of the social or economic stressor on the outcomes was central to the analysis (i.e., not simply included as a covariate to adjust for confounding).

### Cardiometabolic Outcome Measures

We considered three categories of cardiometabolic indicators: (1) carbohydrate metabolism-related biomarkers, including acute and integrated markers of diabetes risk (e.g., glucose, HbA1c, insulin resistance); (2) common lipid outcomes known to be associated with cardiometabolic risk, including total cholesterol, low density lipoprotein (LDL), high density lipoprotein (HDL), cholesterol, triglycerides, apolipoproteins A and B; and (3) central adiposity (e.g., waist circumference (WC), waist-hip ratio (WHR), waist standard deviation). We did not include metabolic syndrome, because it lacks consensus on definition and are not uniformly accepted as valid within pediatric and adolescent populations [29].

### Socioeconomic and Other Social Stressors

In the absence of an explicit operational definition of social and economic stressors for child development research, we relied on a definition of “social context” articulated by Boyce and colleagues [30], which defines social context as “a set of interpersonal conditions, relevant to a particular behavior or disorder and external to, but shaped and interpreted by, the individual child” (p. 146). In line with this broad definition, our review sought to include studies that have considered measures of contextual and material hardships, relative disadvantage, family SES, stressful experiences, and relationship stressors (i.e., with parents or peers).

### Search Strategy and Data Extraction

We conducted systematic searches of PubMed and ISI Web of Science (including Science Citation Index Expanded, Social Sciences Citation Index, and Arts and Humanities Citation Index) to identify relevant studies published in English between January 2001 and January 2013. Our Pubmed search was guided by Medical Subject Heading terms and keywords, including but not limited to: *body fat distribution, waist-hip ratio, blood glucose, lipids/blood, insulin resistance, socioeconomic factors, social environment* and *interpersonal relations* (see Appendix S1); this search returned 1304 abstracts. A similar strategy was developed for ISI Web of Science, and this search returned 773 abstracts. After we removed duplicate abstracts (n = 351), we screened each abstract according to the four criteria outline above. Of note, we carefully examined studies that focused on composite outcomes (e.g., allostatic load, metabolic syndrome, insulin resistance syndrome) in order to establish whether the study reported associations for component factors as well; if so, the study was eligible for inclusion. After applying our criteria, the PubMed and ISI Web of Science searches yielded 36 relevant studies, and one additional study was identified within a reference section of an identified article (see Figure S1). We reviewed these 37 studies and extracted information related to design, sample, measures, statistical methods, stratification and control variables, and findings. The reported findings are based on models adjusted for standard covariates, including age, sex, and race/ethnicity, when provided, and when effect modification was considered, we include that information.

### Evidence Grade

We assessed the strength of the evidence by rating four components of each study's methodology, including study design, sample size, covariates, and exposure measures. For study design, we awarded one point for longitudinal or prospective designs (i.e., repeated measures on the same individual, or following an individual over time with a time lag between the exposure and outcome). For sample size, we awarded one point to studies that had an *n* greater than 500 [31]. For covariates, we awarded one point to studies that provided results adjusted for at least basic demographics including age and sex. Finally, if a study examined more than one exposure measure (e.g., SES measured using both parental income and education), we awarded one point, as this provided more information about the consistency and generalizability of associations. Studies that received at least three points were identified as “high quality” for the purpose of this review.

## Results

Tables 1 and 2 summarize the 37 publications (originating from 31 samples), organized by outcomes. Most studies (32 of 37) examined at least one SES exposure. Table 1 presents studies with SES-related exposures, and Table 2 presents studies with non-SES exposures (“other social stressors”), which may or may not be influenced by SES.

**Table 1:** Studies examining socioeconomic status and cardiometabolic biomarkers in youth, January 2001 through January 2013.

	Country; Study name if >1 article	Design, *n*	Ages a	Stressor ^b^	Outcomes	Findings ^c^	Expected direction?	Evidence Grade ^d^
*Carbohydrate Metabolism*								
Ali et al., 2011	USA; NHANES 1999–2008	Cross-sectional, *n* = 16,085	6–17	Poverty-income ratio	HbA1c	Null.	No	++
Buchan et al., 2012	Scotland	Cross-sectional, *n* = 107	16.4 (± 0.7)	Free school meal eligibility; Scottish Index of Multiple Deprivation	Insulin; glucose	Among boys, lower SES was associated with higher glucose. Among girls, lower SES was associated with higher glucose and lower insulin.	Mixed, based on outcome; conditional, by sex	+
Eldeirawi & Lipton, 2003	U.S.A.; NHANES 1988–1994	Cross-sectional, *n* = 4928	4–17	Poverty-income ratio	HbA1c	Null.	No	++
Goodman et al., 2005	U.S.A; Princeton School District Study	Cross-sectional, *n* = 758	13–19	Highest parental education	Insulin; glucose; HbA1c; insulin resistance	Lower education associated with higher insulin, higher glucose, and greater insulin resistance.	Mixed, based on outcomes	++
Goodman et al., 2007	U.S.A; Princeton School District Study	Longitudinal, *n* = 1167	13–19	Highest parental education; income	Insulin resistance	Lower education associated with baseline insulin resistance, and worsening insulin resistance over time; effect especially strong for obese youth.	Mixed, based on exposures	++++
Goodman et al., 2010	U.S.A; Princeton School District Study	Longitudinal, *n* = 1222	13–19	Highest parental education	Insulin	Education associated with higher insulin at follow-up, adjusting for baseline.	Yes	+++
Gower et al., 2003	U.S.A	Longitudinal, *n* = 125	5–16	Hollingshead index	Insulin; insulin sensitivity; acute insulin response to glucose	SES associated with acute insulin response to glucose.	Mixed, based on outcomes	++
Lawlor et al., 2005	Denmark, Estonia, Portugal	Cross-sectional, *n = *3189	9–15	Maternal and paternal education; income	Insulin resistance	Varied by country: Danish children from poorer and less educated families had greater insulin resistance; in Estonia and Portugal, children from poorer and less educated parents had lower insulin resistance.	Conditional, by country	+++
Thomas et al., 2012	England	Cross-sectional, *n* = 4804	9–11	Highest parental occupation	HbA1c; glucose; insulin resistance	In White students, lower occupation was associated with greater insulin resistance; in Black students, lower occupation was associated with lower insulin resistance (no associations for South Asians).	Conditional, by race; and, mixed based on outcome	++
van den Berg et al, 2012	The Nether-lands	Cross-sectional, *n* = 1308	5–6	Maternal education; self-report income adequacy	Glucose; insulin resistance	Low maternal education was associated with higher glucose and insulin resistance.	Mixed, based on exposure	+++
Wennlof et al., 2005	Sweden	Cross-sectional, *n* = 969	9–15	Maternal education	Insulin; glucose	Null.	No	++
*Lipids*								
Alberty et al., 2009	Slovakia	Cross-sectional, *n = *788	7–17	Income	Fasting TC minus HDL	Greater household income positively associated with greater non-HDL cholesterol.	No	++
Ali et al., 2011	USA	Cross-sectional, *n* = 16,085; NHANES 1998–2008	6–17	Poverty-income ratio	TC minus HDL (fasting status not specified)	Null.	No	++
Buchan et al., 2012	Scotland	Cross-sectional, *n* = 107	16.4 (± 0.7)	Free school meal eligibility; Scottish Index of Multiple Deprivation	Fasting HDL; LDL	Null.	No	+
Goodman et al., 2005	U.S.A; Princeton School District Study	Cross-sectional, *n* = 758	13–19	Highest parental education	Fasting HDL; LDL; TG	Lower education associated with higher LDL and lower HDL.	Mixed, based on outcome	++
Howe et al., 2010	England; ALSPAC	Cross-sectional, *n* = 7772	10	Maternal education	Non-fasting TC; HDL; TG; apolipoproteins A and B	Education was associated with apolipoprotein B.	Mixed, based on outcome	++
Kant et al., 2012	USA; NHANES 2003–2006	Cross-sectional, *n* = 2700	2–19	Poverty-income ratio; education of head of household	Fasting TC; HDL; LDL; TG	Null.	No	+++
Khanolkhar et al., 2012	Sweden	Cross-sectional, *n* = 1204	5–14	Maternal and paternal education; maternal and paternal occupational class	TC; ratio of apolipoproteins A and B (fasting status not specified)	Few inconsistent associations were observed for both TC and ratio of apolipoproteins A and B for both maternal and paternal occupational class.	Mixed, based on exposure	+++
Kvaavik et al., 2012	Norway	Prospective,*n* = 498	11–15	Maternal and paternal education	TC; TG (fasting for some participants)	Null.	No	+++
McCrindle et al., 2010	Canada	Cross-sectional, *n* = 20719	14–15	School district income	Non-fasting TC	Null.	No	++
Murasko, 2008	U.S.A.; NHANES 1999–2004	Cross-sectional, *n* = 4788 (HDL), *n = *2137 (LDL)	12–17	Income	HDL; LDL (fasting for some participants)	Greater household income associated with reduced probability of low HDL, and association more pronounced for females.	Mixed, based on outcome	++
Thomas et al., 2012	England	Cross-sectional, *n* = 4804	9–11	Highest parental occupation	Fasting TG; HDL	In White students, lower SES was associated with higher TG; in Black students, lower SES was associated with lower TG.	Conditional, by race; mixed, based on exposure	++
van den Berg et al, 2012	The Nether-lands	Cross-sectional, *n* = 1308	5–6	Maternal education; self-report income adequacy	Fasting TC; HDL; TG	Null.	No	+++
Van Lenthe et al., 2001	Ireland	Prospective, *n* = 509	12	Occupation	Non fasting TC; HDL; TC/HDL	Among boys at age 15 (but not girls), HDL was greater among youth with parents that had manual occupations, and TC/HDL was lower in this group.	No	+++
Wennlof et al., 2005	Sweden	Cross-sectional, *n* = 969	9–15	Maternal education	Fasting TC; HDL; TG	Null.	No	++
*Central Adiposity*								
Ali et al., 2011	USA; NHANES 1998–2008	Cross-sectional, *n* = 16,085	6–24	Poverty-income ratio	Waist-to-height ratio>0.5	Among boys ages 6–11 and girls ages 12–17, lower poverty-income ratio was associated with higher prevalence of central obesity.	Conditional, by sex and age	++
Bjelland et al., 2010	Norway	Cross-sectional, *n* = 1483	11	Highest parental education	WC; WHR	Lower education associated with higher WC and WHR.	Yes	++
Brown et al., 2012	U.S.A.	Cross-sectional, *n = *125	5.6 (kinder-tarden) and 8.7 (3^rd^ grade)	Maternal and paternal education	WC; WHR	Among 3^rd^ grade girls, lower paternal education was associated with higher WC and WHR.	Conditional, by sex; mixed, based on exposure	++
Brug et al., 2012	Belguim, Greece, Hungary, Nether-lands, Norway, Slovenia, Spain	Cross-sectional, *n* = 7234	10–12	Highest parental education	WC	Across countries, lower parental education was associated with higher WC.	Yes	+
Buchan et al., 2012	Scotland	Cross-sectional, *n* = 107	16.4 (± 0.7)	Free school meal eligibility; Scottish Index of Multiple Deprivation	WC	Null.	No	+
Goodman et al., 2005	U.S.A.; Princeton School District Study	Cross-sectional, *n* = 758	13–19	Highest parental education; income	WC	Lower education associated with higher WC.	Mixed, based on exposure	+++
Jimenez-Pavon et al., 2010	Spain	Cross-sectional, *n* = 1795	12.5–18.5	Maternal and paternal education; occupation	WC	Higher education was associated with lower WC in boys but not girls; no association for profession status.	Conditional, by sex; mixed, based on exposure	+++
Kendzor et al., 2012	U.S.A.	Prospective, *n* = 1356	15	Household income trajectory from birth to 15	WC	Downward income trajectory and stable low income from birth to age 15 were associated with greater WC.	Yes	+++
Koziel & Jankowska, 2002	Poland	Cross-sectional, *n* = 2016	14	Maternal education	WHR	Lower education associated with higher WHR among girls (not boys).	Conditional	++
Moore et al., 2002	U.S.A.	Longitudinal, *n* = 235	8.8 (±2)	Hollingshead index	WC; WHR	Lower SES associated with greater increase in WC over time.	Mixed, based on outcome	+
Ness et al., 2006	England; ALSPAC	Prospective, *n* = 5917	9.9 (± 0.33)	Lowest parental social class	Trunk fat (kg)	Null.	No	+++
Ortega et al., 2012	Estonia, Sweden	Longitudinal, *n* = 949	9–15	Maternal education	WC	High maternal education was associated with decreased odds of remaining in the top quartile of WC over the 6 years follow-up.	Yes	+++
Okosun et al., 2006	U.S.A.	Cross-sectional, *n* = 5020	6–11	Highest parental education	WC	Lower education associated with higher probability of WC >95^th^ percentile.	Yes	++
Thomas et al., 2012	England	Cross-sectional, *n* = 4804	9–11	Highest parental occupation	WC	Among White students, lower SES was associated with greater WC.	Conditional, by race	++
Wake et al., 2007	Australia	Cross-sectional, *n* = 4938	4–5	Maternal education; occupation; income; area-level disadvantage	WC	Null.	No	+++
Wardle et al., 2006	England	Longitudinal, *n* = 5863	11–12	Area-level deprivation	WC; waist standard deviation	Higher area-level socioeconomic deprivation associated with trajectory of WC and waist standard deviation.	Yes	+++
Yin et al., 2005	U.S.A.	Cross-sectional, *n* = 303	12–24	Community-level economic disadvantage	WC	Community disadvantage associated with higher WC.	Yes	++

a Age at baseline outcome measurement;^ b^ Refers to parent SES status; ^c^ Only significant findings are reported; describes adjusted model findings, if provided (e.g., control variables of age, sex, race/ethnicity). ^d^ The strength of the evidence was evaluated based on four components of each study's methodology, including study design, sample size, covariates, and exposure measures. LDL = Low density lipoprotein cholesterol; HDL = High density lipoprotein cholesterol; TG = Triglycerides; TC = Total cholesterol; Apo = Apolipoprotein; WC = Waist circumference; WHR = Waist-hip ratio; ALSPAC = Avon Longitudinal Study of Parents and Children.

**Table 2:** Studies examining social stressors and cardiometabolic biomarkers in youth, January 2001 through March 2012.

	Country	Design, *n*	Ages a		Stressor	Outcomes	Findings ^b^	Expected direction?	Evidence Grade ^d^
*Carbohydrate Metabolism*									
Marin et al., 2007	Canada	Cross-sectional, *n* = 104	15–19		Stressful events; interpersonal stress	Insulin; glucose	Null.	No	+
Ravaja, N., et al. (2001).	Finland	Longitudinal, n = 451	9 years		Self-rated maternal child rearing	Insulin	Among girls (but not boys), mother's low tolerance towards the child predicted higher insulin.	Conditional, by sex	++
*Lipids*									
Buchmann et al., 2010	Germany	Prospective, *n* = 207	19	Rearing practices; maternal responsiveness		Fasting HDL; LDL; TG; TC; Apo A1, B C3, and E	Adverse rearing and poor responsiveness associated with lower HDL and apolipoprotein A1.	Mixed, by outcome	+++
Ravaja, N., et al. (2001).	Finland	Longitudinal, n = 451	9 years	Self-rated maternal child rearing		Fasting HDL; triglycerides	Among boys (but not girls), hostile maternal child-rearing attitudes predicted HDL. Among girls (but not boys), strict disciplinary style of the mother predicted higher TG.	Conditional, and mixed by outcome	++
*Central Adiposity*									
Buchmann et al., 2001	Germany	Prospective, *n* = 207	19	Rearing practices; responsiveness		WHR	Null.	No	+++
Kim et al., 2008	U.S.A.	Cross-sectional, *n* = 106	13–15	Maternal and paternal rearing practices		WC	Maternal authoritative style associated with smaller WC; maternal control associated with greater WC.	Mixed, by exposure.	++
Midei & Matthews, 2009	U.S.A.	Longitudinal, *n* = 213	14–16	Lack of supportive relationships		WHR	Fewer supportive relationships predicted increased WHR over time.	Yes	++
Yin et al., 2005	U.S.A.	Cross-sectional, *n* = 303	12–24	Stressful events		WC	Stressful life events associated with higher WC.	Yes	++

a Age at baseline outcome measurement;^ b^ Only significant findings are reported; describes adjusted model findings, if provided (e.g., control variables of age, sex, race/ethnicity). ^d^ The strength of the evidence was evaluated based on four components of each study's methodology, including study design, sample size, covariates, and exposure measures. LDL = Low density lipoprotein cholesterol; HDL = High density lipoprotein cholesterol; TG = Triglycerides; TC = Total cholesterol; Apo = Apolipoprotein; WC = Waist circumference; WHR = Waist-hip ratio.

### Socioeconomic Status-Related Exposures and Cardiometabolic Risk Markers

#### Carbohydrate Metabolism-related Outcomes

Table 1 provides a summary of the eight cross-sectional and three prospective studies of SES and carbohydrate metabolism-related outcomes, including insulin, glucose, HbA1c, insulin sensitivity, acute insulin response to glucose, and insulin resistance. Six of the eleven studies evaluated more than one relevant outcome, and the most common SES measure used was parental education. All eleven studies reported findings adjusted for basic covariates (i.e., at a minimum, age and sex). Overall, the findings lack consistency. Of the eleven studies, three were null [32]–[34], one study found an association in the expected direction (only one exposure and one relevant outcome considered) [35], two studies had conditional findings (whereby the direction of associations varied by country [36] and race [37]) and six studies had mixed findings (three resulted from different associations of the same measure of SES with two different outcome measures, and the other three resulted from discrepant findings with different measures of SES and the same outcomes). When we consider the 3 prospective studies on their own [35], [38], [39], the findings are more consistent (i.e., none of these studies had null findings) and provide some evidence for an association between socioeconomic disadvantage and elevated risk. Four of the eleven studies that examined carbohydrate metabolism-related factors were classified as higher-quality based on our evidence rating [35], [36], [38], [40], and each provided some positive evidence for an association.

#### Lipid Outcomes

Fourteen studies examined SES in relation to lipid outcomes (i.e., total cholesterol, LDL and HDL cholesterol, triglycerides, apolipoproteins A and B); twelve of these studies were cross-sectional and two studies were prospective [41], [42]. Most studies considered a single SES exposure, however the majority evaluated more than one lipid outcome. All fourteen studies reported results adjusted for basic covariates (i.e., at a minimum, age and sex). These studies do not indicate a consistent association between SES and lipid outcomes among youth. Of the fourteen studies, seven were null for all associations that were examined [32], [34], [40], [41], [43]–[45], and two showed associations in the direction opposite to the expected direction [42], [46]. Considering the other five studies, four had had mixed results due to differences in the association of SES with multiple lipid outcomes [37], [47]–[49], one had mixed results due to discrepant findings resulting from different measures of SES [50], and in one of these studies, the mixed results also varied by race [37]. Of note, there were not observable patterns across the studies that produced mixed results. Five of the fourteen studies that examined SES in relation to lipid outcomes were rated as higher quality [34], [41], [42], [44], [50], and four of these studies had null results.

#### Central Adiposity

Our search identified twelve cross-sectional and five prospective studies which examined SES with central adiposity (measured by WC, WHR, waist standard deviation, and trunk fat (kg)). The majority of these studies incorporated only one measure of SES, and parental education was the most common measure. Sixteen of the seventeen studies reported associations from models adjusted for basic covariates (i.e., at a minimum, age and sex). Although the studies are not entirely consistent, they suggest an inverse relationship between SES and central adiposity in youth. Seven of the seventeen studies found that lower SES was associated with greater central adiposity [51]–[57]. Four studies showed conditional associations by sex (with no consistent pattern) [32], [58]–[60], one study found results conditional by race [37], and four studies had mixed findings [47], [58], [59], [61]; one mixed finding was due to inconsistent associations of SES with WC versus WHR, and three mixed findings were due to inconsistent associations across different SES measures with the same outcome. Only three studies were null for all associations that were examined [43], [62], [63]; notably, all of the null studies used non-US samples. Of the five prospective studies, three studies showed that lower SES was associated with greater central adiposity, one study showed no association between SES and central adiposity, and the other study had mixed results whereby lower SES was associated with greater increases in WC (but not WHR) over time. Eight studies were classified as higher-quality; among these studies, six studies showed at least some evidence of an association [37], [47], [53], [54], [56], [59] (three of these studies had mixed results due differences across measures or were conditional on sex or race [37], [47], [59]), and two studies were null [62], [63].

### Other Social Stressors and Cardiometabolic Risk Markers

Our search identified six studies that examined a possible relationship between parenting practices, stressful life events or relational support and our cardiometabolic risk markers (see Table 2). Half of these studies were prospective, as opposed to the preponderance of cross-sectional studies on SES noted above, and the sample sizes were smaller (range: N = 104–451, median N = 210). Roughly half of these studies adjusted for SES while examining the associations of other social stressors to cardiometabolic risks. Five of the six studies reported associations adjusted for basic covariates (i.e., at a minimum, age and sex). With only one or two studies assessing similar exposures and outcomes, it is not possible to assess patterns of associations between these other types of social stressors and our cardiometabolic risks. Two studies considered associations of relevant social stressors (stressful life events and interpersonal stress) in relation to carbohydrate metabolism-related factors: one documented null associations between stressful events and interpersonal stress with insulin and glucose among female adolescents [64], and the other found that mother's low tolerance towards the child predicted higher insulin among girls but not boys [65]. Two studies considered parental rearing practices and maternal responsiveness in relation to multiple lipid outcomes, with mixed results based on outcome [65], [66] and sex [65]. Of the four studies that considered central adiposity, these considered parental responsiveness, rearing practices, lack of supportive relationships, and stressful life events as the stressors, with the latter 3 showing associations with outcomes in the expected direction. Across all six studies, the findings were similarly inconsistent for prospective and cross-sectional studies. Only one study was rated as higher-quality [66]; this prospective study found mixed support for an association between child rearing practices and maternal responsiveness and lipid outcomes, and a null association with central adiposity.

## Discussion

Given increasing understanding that a child's early experiences has profound effects on risk for chronic diseases later in life [6], [67] and the escalating societal burden of cardiovascular [68] and metabolic [69] diseases in the United States, it is important to identify how and when disease processes are initiated to develop effective prevention and early intervention strategies. In this systematic review, we identified 37 published articles of socioeconomic disadvantage and other social stressors in relation to three potential mechanisms that may connect early socioeconomic disadvantage and other social stressors to adult cardiometabolic disease: carbohydrate metabolism-related factors, lipids, and waist circumference. The clearest evidence emerged for the relation between socioeconomic disadvantage and central adiposity, which is consistent with findings from recent reviews of childhood experiences and overweight and obesity [13]–[15]. While this finding is important given that central fat may be particularly harmful for long-term health [70], it is not unexpected and unfortunately does not shed new light on other pathways by which social stressors may contribute to development of cardiometabolic diseases. In fact, what this review most clearly demonstrates is that surprisingly, research on the relationship between stressors and carbohydrate and lipid metabolism-related risks is too sparse to be able to form strong conclusions, particularly with regard to non-SES social exposures. Further, the few published studies we found rarely assessed identical exposures and outcomes or used a similar study design. A review of only the prospective or higher-quality studies also showed inconsistent associations between socioeconomic or other social stressors and carbohydrate and lipid metabolism-related factors, without any discernible patterns that could explain the discrepant associations.

While we found relatively few studies on social stressors and carbohydrate and lipid-related risks, considerable research has examined stressful social environments in relation to cognitive, behavioral, and other physical health outcomes in children [71], particularly overweight and obesity [14], [15], [72]–[75]. Although obesity is an important risk factor to consider, indicators from other physiological parameters that may respond to stress are worthy of investigation because they may provide additional insight on the mechanisms that underlie cardiometabolic disorders [76]. Increasing research has documented that the distribution of body fat contributes to diabetes and cardiovascular risk among adults independent of general assessments of adiposity [70], [77]. Other research using NHANES participants aged 12–19 found that in linear regression models adjusted for age, survey period, and race-ethnicity, body fat percentage only explained 2–20% of the variance in lipid concentrations [78]. Such findings suggest that it is important to examine the relationship between stressful experiences and cardiometabolic risk factors beyond basic consideration of adiposity in youth. Research on how socioeconomic disadvantage and other social stressors affect a variety of cardiometabolic risk markers early in life will improve our understanding of how stress experiences become biologically embedded and lead to metabolic alterations and weight change, and may elucidate new pathways and opportunities for earlier interventions to prevent cardiometabolic disorders.

Comparison of the findings from the present review to previous reviews considering similar exposures (social disadvantage and other social stressors) in relation to overweight and obesity [14], [15], [72], [73] and inflammatory biomarkers [75] suggests that studies on the outcomes we consider are fewer and also less consistent. For example, in a review of 45 studies from developed countries (1990 to 2005), Shrewsbury and colleagues [72] found inverse associations between SES and adiposity in 42% of studies, mixed or conditional associations in 31%, and null associations in 27%. These associations were most consistent when parental education was used as the indicator of SES (i.e., 75% of studies that examined education as the exposure found an inverse association). In our review, education was not more consistently associated with outcomes relative to other SES indicators. However, because only a few studies examined parental educational attainment in relation to each specific outcome, additional research is needed to determine if education is in fact a particularly strong predictor of these cardiometabolic factors as well. Carter and colleagues [15] examined 27 studies (1999 to 2009) of the relationship between neighborhood characteristics and child adiposity. Across studies, area-level socioeconomic disadvantage was positively associated with adiposity, and there was some evidence that greater social capital was inversely associated with adiposity [15]. In our review, only 3 studies considered area-level environmental features [45], [56], [57] (and 2 of the 3 examined central adiposity [56], [57]); therefore we do not have enough studies to determine whether area-level measures are consistent predictors of other cardiometabolic outcomes.

There has been increasing interest in whether childhood adversity influences risk of low level chronic inflammation [79]–[81], with more studies focusing on CVD-relevant inflammatory and other immune-related biomarkers in youth relative to those focusing on lipids or carbohydrate metabolism-related factors. Inflammatory processes have been identified as another plausible mechanism by which socioeconomic disadvantage and other social stressors increase later risk for cardiometabolic diseases [7]. A recent systematic review of 20 published studies of social adversity and inflammation in youth suggests a trend towards positive associations [75]. At present, it is unclear whether heightened inflammatory markers in response to childhood adversity appear earlier in development compared to elevations in carbohydrate metabolism-related factors or lipids (which may become evident later, perhaps as a downstream consequence of adiposity). Additional studies are needed in order to establish whether the different strength of findings across domains of outcomes (i.e., adiposity, inflammation, carbohydrate metabolism-related markers, lipids) are a function of more limited research available on carbohydrate metabolism-related markers and lipids or because in fact these alterations are less evident early in life.

Our review suggests a number of priorities for future research. First, our review reveals a striking paucity of longitudinal studies to examine the effects of socioeconomic disadvantage and other social stressors on carbohydrate metabolism-related factors, lipids, and central adiposity. From cross-sectional studies, it is not possible to assess when cardiometabolic risk factors begin to emerge in response to social disadvantage or other stressors. Therefore, the next generation of life course research aiming to identify social and biological mechanisms by which socioeconomic disadvantage and other social stressors are embedded to influence adult health, will require investment in longitudinal cohorts with extensive data collection on social conditions and experiences and health outcomes at multiple time points. Although longitudinal studies are more time-intensive and expensive than cross-sectional studies, they address concerns about the temporal ordering between exposures and outcomes, and provide insight into whether there are particular periods of development when these cardiometabolic biomarkers are especially sensitive to, or resilient against, certain social exposures. Longitudinal studies will further allow investigators to identify if effects of social stress depend on developmental stage (i.e., sensitive periods) and at what point in the life course they are detectable.

Second, our review shows there are many types of social stressors (e.g., child maltreatment, parent psychopathology, parental intimate partner violence) that have not been examined in relation to the markers considered in this review, but that have shown to be relevant to other physiological outcomes (such as BMI [82] or inflammation [79], [80]) in youth. Thus, there is a need for future studies to assess a wider variety of types and severity (ranging from minor to severe, acute and chronic) of social stressors and compare effects within the same sample, to identify which are most toxic in relation to cardiometabolic factors. For example, a review by Berge and colleagues [73] reported substantial evidence that parenting style is associated with child BMI [73]. However, our review only identified one study that (prospectively) examined parenting style in relation to insulin and lipids [65]. Therefore, additional research that considers parenting style and other types of social stressors, in relation to a broader more diverse set of cardiometabolic risk markers would be fruitful.

Third, several researchers have begun to examine childhood stressors in relation to cumulative biological risk scores (e.g., allostatic load) [47], [83]–[85] and cardiovascular risk phenotypes (e.g., metabolic syndrome [86]) in youth. These approaches may be valuable for identifying meaningful dysregulation when the effect of an exposure on one specific biomarker is small or inconsistent, but there is a distinguishable effect when you consider a number of related physiological indicators. Additional research is needed to assess whether composite approaches (incorporating individual or multiple systems) within pediatric populations are meaningful for long-term health outcomes, and if composite approaches provide any advantages for understanding the effects of early adversity for later risk of cardiometabolic disorders.

It is important to acknowledge several limitations to the present review. First, several studies used the same sample to examine more than one type of outcome, or the same outcome at a later time point; this could make the literature appear to be more consistent than it actually is. Related, several studies that considered more than one outcome did not calculate a family-wise error, which may compromise the validity of the statistical associations we report. However, in light of the sparse research in this area, we included all unique findings that exist. Finally, this review is limited to studies published in English, and we cannot account for publication bias towards studies with significant results.

In conclusion, scientific understanding of the biological pathways that connect early life experiences to cardiometabolic risk in adulthood remains limited. With improved understanding of the relationship between social adversity and less-studied cardiometabolic risk factors such as glucose, insulin, and lipids among youth, we may begin to identify key intervention opportunities to protect the health of children and adolescents, and the adults they will become.

## Supporting Information

Figure S1 Prisma 2009 Flow Diagram
(TIF)

Appendix S1 Pubmed Search Strategy.
(DOC)

Appendix S2 Prisma 2009 Checklist.
(DOC)
