# Supplementary figures and images for: Socioeconomic and Other Social Stressors and Biomarkers of Cardiometabolic Risk in Youth: A Systematic Review of Less Studied Risk Factors
Source: PLoS One. 2013 May 17;8(5):e64418. doi: 10.1371/journal.pone.0064418 (PMC3656855; doi:10.1371/journal.pone.0064418)

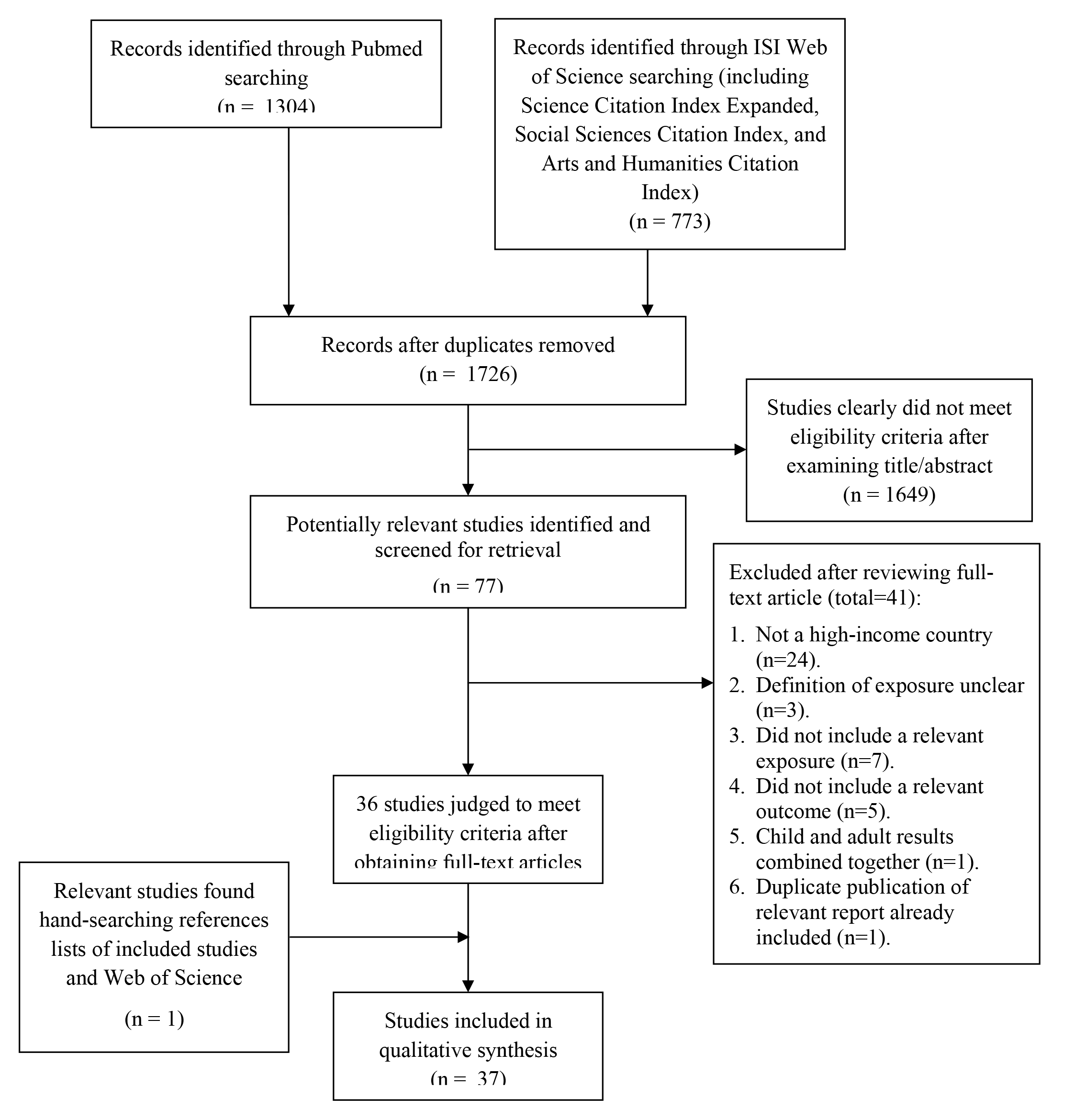

Supplement: Figure S1 — Prisma 2009 Flow Diagram (TIF) [file pone.0064418.s001.tif]
