# Supplementary material for: Socioeconomic and Other Social Stressors and Biomarkers of Cardiometabolic Risk in Youth: A Systematic Review of Less Studied Risk Factors
Source: PLoS One. 2013 May 17;8(5):e64418. doi: 10.1371/journal.pone.0064418 (PMC3656855; doi:10.1371/journal.pone.0064418)
Supplement: Appendix S1 — Pubmed Search Strategy. (DOC) [file pone.0064418.s002.doc]

**Appendix S1. Pubmed Search Strategy**

("Body Fat Distribution"[Mesh] OR "Waist-Hip Ratio"[Mesh] OR "Blood Glucose"[Mesh] OR "Insulin/blood"[Mesh] OR "Hemoglobin A, Glycosylated/blood"[Mesh] OR "Lipids/blood"[Mesh] OR “Triglycerides/blood”[Mesh] OR "Body Fat Distribution"[ tiab] OR "Waist-Hip Ratio"[ tiab] OR "Blood Glucose"[ tiab] OR "Insulin"[ tiab] OR "Glycosylated Hemoglobin A"[ tiab] OR "Lipids"[ tiab] OR “Triglycerides”[tiab] OR "waist circumference"[tiab] OR "central adiposity"[tiab]) AND ("Interpersonal Relations"[Mesh] OR "Stress, Psychological"[Mesh] OR "Socioeconomic Factors"[Mesh] OR "Life Change Events"[Mesh] OR "Social Environment"[Mesh] OR "Family Relations"[Mesh] OR "Conflict (Psychology)"[Mesh] OR "Aggression"[Mesh] OR "Violence"[Mesh] OR "Prejudice"[Mesh] OR “Interpersonal Stress”[tiab] OR “social stress”[tiab] OR “socioeconomic”[tiab] OR “stressful life”[tiab] OR “family climate”[tiab] OR “maternal education”[tiab] OR “economic resources”[tiab] OR “income”[tiab] OR unemployment[tiab] OR conflict[tiab] OR “attachment”[tiab] or “parenting style”[tiab]) AND ("Infant"[Mesh] OR "Child"[Mesh] OR "Adolescent"[Mesh] OR "child"[tiab] OR "adolescent"[tiab] OR "youth"[tiab]) AND English[lang] AND "2001"[Publication Date] : "2013"[Publication Date]
